# Eddy Current Sensor System for Blade Tip Clearance Measurement Based on a Speed Adjustment Model

**DOI:** 10.3390/s19040761

**Published:** 2019-02-13

**Authors:** Jiang Wu, Bin Wen, Yu Zhou, Qi Zhang, Shuiting Ding, Farong Du, Shuguang Zhang

**Affiliations:** 1 School of Transportation Science and Engineering, Beihang University, Beijing 100191, China; wu_jiang@buaa.edu.cn (J.W.); SY1613213@buaa.edu.cn (B.W.); gnahz@buaa.edu.cn (S.Z.); 2 Beijing Key Laboratory for High-efficient Power Transmission and System Control of New Energy Resource Vehicle, Beihang University, Beijing 100191, China; 3 School of Energy and Power Engineering, Beihang University, Beijing 100191, China; dst@buaa.edu.cn (S.D.); dfr@buaa.edu.cn (F.D.)

**Keywords:** blade tip clearance (BTC), eddy current sensor (ECS), measurement method, nonlinear regression, speed adjustment model (SAM)

## Abstract

Blade tip clearance (BTC) measurement and active clearance control (ACC) are becoming crucial technologies in aero-engine health monitoring so as to improve the efficiency and reliability as well as to ensure timely maintenance. Eddy current sensor (ECS) offers an attractive option for BTC measurement due to its robustness, whereas current approaches have not considered two issues sufficiently. One is that BTC affects the response time of a measurement loop, the other is that ECS signal decays with increasing speed. This paper proposes a speed adjustment model (SAM) to deal with these issues in detail. SAM is trained using a nonlinear regression method from a dynamic training data set obtained by an experiment. The Levenberg–Marquardt (LM) algorithm is used to estimate SAM characteristic parameters. The quantitative relationship between the response time of ECS measurement loop and BTC, as well as the output signal and speed are obtained. A BTC measurement method (BTCMM) based on the SAM is proposed and a geometric constraint equation is constructed to assess the accuracy of BTC measurement. Experiment on a real-time BTC measurement during the running process for a micro turbojet engine is conducted to validate the BTCMM. It is desirable and significative to effectively improve BTC measurement accuracy and expand the range of applicable engine speed.

## 1. Introduction

With increasing applications of turbomachinery in various industrial fields, the requirement for multi-objective design methods, such as thermal efficiency, aerodynamic performance and structural life, has considerably increased in the last few decades [1,2,3]. As one of the key constraints in blade design, the blade tip clearance (BTC) changes in a complicated manner under various working statuses of rotors. Actually, reasonable BTC is beneficial for the performance and reliability of turbomachinery. The relatively large BTC will enhance the heat transfer in the blade tip region of turbomachinery, exacerbate the oxidation and erosion of the material for blade tip, and cause performance deterioration or even failure, which accounts for about a third [1] of the failure probability of high pressure turbine; conversely, the relatively small BTC may bring a friction risk between the blade and the cabinet [4]. In addition, unreasonable BTC decreases the thermal efficiency of turbomachinery, for instance, the aerodynamic loss caused by leakage flow of blade tip from the pressure surface to the suction surface accounts for about a third [5] of the total loss for a turbomachinery. Therefore, BTC monitoring can be considered as one of the effective methods of mechanical health management for turbomachinery [6,7,8,9]. Furthermore, the active control of BTC is conductive to improve the aerodynamic efficiency and avoid the risk of blade failure [10,11,12].

To monitor or control BTC, the key lies on real-time dynamic measurement for BTC. However, the minimal clearance existing between the moving blades with high speed and the static cabinet is not able to be directly measured by conventional approaches. Recently, quite a number of novel methods for fast and accurate BTC measurement have been developed, such as optical methods [13,14,15,16] based on the intensity modulation of the reflected light, capacitance method [17,18,19,20] by constructed capacitance between the cabinet and blade tip, eddy current method [21,22,23] by installing the sensor coil probe in the cabinet to form an eddy current on the blade tip surface, and the microwave method [24,25,26] based on the principle, which is similar to a short range radar system. At present, optical methods and capacitance methods are widely used [27,28,29,30], generally at the R&D stage of engine. Nevertheless, these two kinds of sensors are easily polluted by dust, water, oil and other dirt, which limits their scope of usage.

Due to the insensitivity to contaminants, eddy current sensors (ECS) reflect stability and reliability pretty well, as well as high measurement accuracy and low cost for operation and maintenance. Hence, it is very suitable for measurement occasions with complicated working environment and inconvenient maintenance [31]. However, there are still some difficulties in adopting ECS for BTC measurement. Firstly, it is difficult to build the mapping relationship between ECS output signal and BTC because the equivalent impedance of the measurement loop are related to not only the impedance of the sensor, but also the geometry of the measured object, the material properties, and the measured gap [31]. Wang [32] derived the derivative of signal amplitude to blade length by designing a simple impeller composed of blades with various lengths, and established the mapping relationship between the ECS output signal and BTC to achieve the BTC measurement. Moreover, working temperature also influences the equivalent impedance of the measurement loop. Han et al. [23] made a temperature adjustment for ECS, which makes it possible for the BTC measurement in the temperature range of 700 K to 1300 K. Afterwards, the size of the measured clearance affects the mutual inductance coefficient between the measured object and the sensor coil, furthermore, influences the equivalent impedance of the measurement loop [31]. This makes that the response frequency of the measurement loop changes with the measured clearance. Actually, as two significant performance indicators, signal-to-noise ratio (SNR) and response frequency are contradictory [21], specifically, more coil turns determine larger signal amplitude and laggard response, fewer coil turns make the SNR unsatisfactory. Consequently, the measured clearance, SNR and response frequency require being considered comprehensively in ECS design. Finally, considerable linear velocity of blade tip requires the ECS response frequency to be high enough, even to MHz level [33], whereas ECS response frequency is lower than that of optical or capacitive sensors, typically lower than 100 kHz [21]. Thus, ECS output signal may attenuate when turbomachinery speed is increased to a certain extent [32].

Therefore, a great challenge exists in seeking a balance between response speed and accuracy so as to measure BTC accurately. Actually, existing investigations on measuring BTC by ECS mostly depend on various tests and have rarely considered problems in the particular case when a rotating blade passes over a sensor; one issue is that BTC affects the response speed of the sensor loop, the other is that the sensor signal decays with speed increasing. The novel aspect of this article is to develop a speed adjustment model to deal with these issues in detail. Experiment on a real-time ECS measurement during the running process for a micro turbojet engine is conducted to validate the model. It is desirable and significative to effectively improve BTC measurement accuracy and expand the range of applicable engine speed. The rest of this paper is organized as follows. A speed adjustment model and a nonlinear regression method based on dynamic training data set are given in Section 2. A BTC measurement method based on the speed adjustment model is proposed in Section 3. Section 4 performs a turbojet-engine test to validate the proposed method. This is followed by the concluding remarks in Section 5.

## 2. Speed Adjustment Model

In this section, the principle and method of BTC measurement using ECS are introduced. Combined with the structure of the turbomachinery, the characteristics of ECS output signal are analyzed, then the speed adjustment model (SAM) and characteristic parameters of the sensor signal are proposed. The SAM is trained using nonlinear regression from a dynamic training data set obtained by the experiment. To solve the nonlinear regression problem, the Levenberg–Marquardt (LM) algorithm is used to estimate the characteristic parameters of the model.

### 2.1. Principle of BTC Measurement by ECS

**Figure 1:**
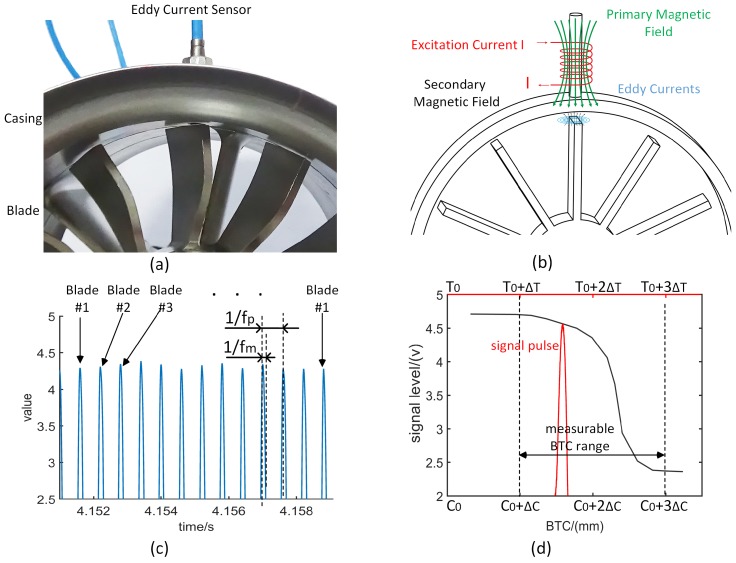
Principle of blade tip clearance (BTC) measurement: (**a**) bladed disk and eddy current sensor (ECS); (**b**) electromagnetic interaction between the blade tip and the sensor (adapted from [31]); (**c**) signal pulse sequence of the sensor corresponding to various blades; (**d**) signal pulse amplitude (SPA) versus BTC ($C_{0}$-initial BTC, ΔC-BTC increment).

In the BTC measurement system based on ECS , the sensor is generally mounted in the casing along the normal direction of the blade tip surface. The sensor probe whose head is parallel to the inner surface of the casing (Figure 1a) has a current of 1–2 MHz in the built-in coil, which forms a primary magnetic field near the tip of the blade. When the rotating blade sweeps over the eddy current probe, a variation of magnetic flux induces eddy currents in the metal surface of the blade tip, which forms a secondary magnetic field and changes the current in the sensor coil (Figure 1b). Because the mutual inductance between the tip of the blade and the coil is related to BTC, the BTC can be measured by detecting and capturing the current change in the coil. In general, the sensor outputs a voltage signal pulse (Figure 1c) when the blade tip rotates past the ECS.

The amplitude of each signal pulse represents the corresponding BTC size of the blade. However, it is not a simple linear relationship between the signal pulse amplitude (SPA) and the BTC. In static state, the BTC–SPA curve can be obtained by measuring the values of SPA under different BTCs (Figure 1d). In practical measurement, according to the collected SPA value, the BTC can be calculated by an interpolation algorithm.The performance of the precision of above methods are satisfactory in the low-speed range. However, the relative-high speed results in that the output signal may deviate from the expected value, which reduces the measurement accuracy.

### 2.2. Speed Adjustment Model

**Figure 2:**
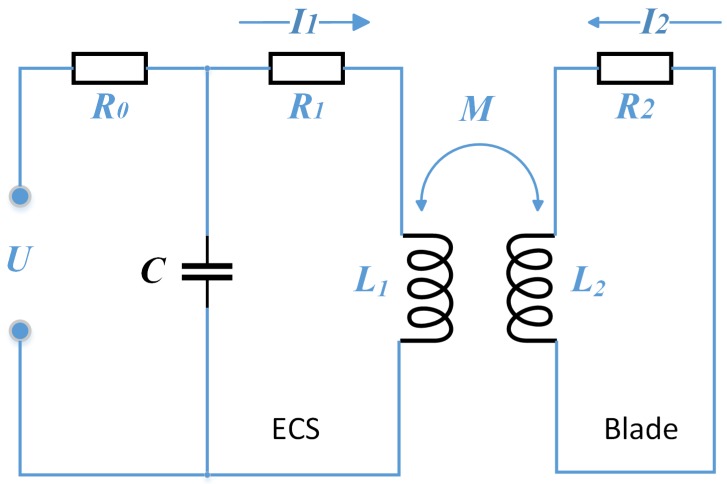
Equivalent circuit of a simple ECS loop and the measured blade.

The mutual inductance *M* between the ECS and the measured blade varies continuously with relative position between them. Assume that *M* remains unchanged, and then analyze the equivalent circuit composed of a simple ECS circuit and the measured blade, as shown in Figure 2. Here, $U_{0}$ is the excitation voltage, $U_{e}$ is the output voltage of detection circuit, $R_{0}$ is the voltage dividing resistance, $R_{e}$ is the equivalent resistance of the ECS, $R_{b}$ is the equivalent resistance of the measured blade surface, $L_{e}$ the equivalent inductance of the ECS, $L_{b}$ is the equivalent inductance of the measured blade surface, *C* is the parallel resonant capacitor, $I_{e}$ is the current in the sensor coil, and $I_{b}$ is the equivalent current of the measured blade surface.

By consideration of the influence of the measured blade on the ECS, the coupled equivalent resistance and inductance of the ECS are as follows [31]:

(1) $$R_{e}^{*}=R_{e}+\frac{{\left(2\pi f\right)}^{2}M^{2}}{{R_{b}}^{2}+{\left(2\pi fL_{b}\right)}^{2}}R_{b},$$

(2) $$L_{e}^{*}=L_{e}-\frac{{\left(2\pi f\right)}^{2}M^{2}}{{R_{b}}^{2}+{\left(2\pi fL_{b}\right)}^{2}}L_{b}.$$

*f* is the excitation frequency. When the ECS is in service, the sensor loop is essentially a second-order LC oscillation circuit. Based on Kirchhoff’s law, we can obtain

(3) $$L_{e}^{*}C\frac{d^{2}U_{e}}{dt^{2}}+R_{e}^{*}C\frac{dU_{e}}{dt}+U_{e}=0.$$

Supposing that the current in the loop is zero when t=0, $U_{e}=U$, then

(4) $$U_{e}=\frac{\omega_{0}}{\sqrt{\omega_{0}^{2}-\delta^{2}}}Ue^{-\delta t}\sin\left(t\sqrt{\omega_{0}^{2}-\delta^{2}}+\beta \right),$$

where $\beta =\arccos(\frac{\delta }{\omega_{0}})$, $0<\beta <\frac{\pi }{2}$, $\delta =\frac{R_{e}^{*}}{2L_{e}^{*}}$, $\omega_{0}=\frac{1}{\sqrt{L_{e}^{*}C}}$. Mark the average of time constant ($\frac{1}{\delta \left(M\right)}$) as $\frac{1}{\bar{\delta }}$; then, the amplitude of $U_{e}$ can be approximated as

(5) $$U_{e}^{A}=\frac{\omega_{0}}{\sqrt{\omega_{0}^{2}-\delta^{2}}}Ue^{-\bar{\delta }t}.$$

The period of interaction between ECS and the blade tip is $t_{m}=\frac{d_{e}}{v_{tip}}$, and the frequency of the interaction is defined (Figure 1c), the measuring frequency $f_{m}$ can be expressed as:

(6) $$f_{m}=\frac{1}{t_{m}}=\frac{\pi nr_{tip}}{30d_{e}},$$

and blade passing frequency [18] $f_{p}$ is given by

(7) $$f_{p}=\frac{n}{60}n_{b},$$

where $t_{m}$ is the measuring time (the duration of the blade tip rotating past the sensor probe), $d_{e}$ represents the coil diameter of a ECS probe, $v_{tip}$ is blade tip velocity, $r_{tip}$ is blade tip radius, *n* is the rotor speed, and $n_{b}$ is the number of blades. $f_{m}$ is the reciprocal of $t_{m}$, and $f_{p}$ is the reciprocal of time interval between adjacent pulses (Figure 1c). In general, the response frequency of the ECS used for BTC measurement is matched to $f_{p}$, but, in fact , $f_{m}$ is much higher than $f_{p}$.

Under the condition that the BTC is an unchanged value $c_{i}$, the SPA $u_{i}$ varies with the rotational speed $n_{i}$. According to Equation (5), the SAM of signal amplitude $u_{i}$ can be expressed as:

(8) $$u_{i}^{\left(c_{i}\right)}=v\left(n_{i},u_{s}^{\left(c_{i}\right)},u_{r}^{\left(c_{i}\right)},\tau^{\left(c_{i}\right)}\right)=u_{s}^{\left(c_{i}\right)}-u_{r}^{\left(c_{i}\right)}e^{-\frac{1\times 10^{4}t_{m}}{\tau^{\left(c_{i}\right)}}}=u_{s}^{\left(c_{i}\right)}-u_{r}^{\left(c_{i}\right)}e^{-\frac{3\times 10^{5}d_{e}}{\pi n_{i}r_{tip}\tau^{\left(c_{i}\right)}}},$$

where $u_{s}$ is the component irrelevant to the rotational speed, $u_{r}$ is the component related to the rotational speed, and τ is the response time of the sensor loop. The constant $1\times 10^{4}$ in the formula is configured to make the order of magnitude of $\partial u/\partial u_{s}$, $\partial u/\partial u_{r}$, ∂u/∂τ more close. As three characteristic parameters of SAM, $u_{s}$, $u_{r}$, τ are pretty essential to describe the variation of SPA with the rotational speed.

The nonlinear regression method [34] can be used to solve the characteristic parameters of SAM, provided that a dynamic training data set **D** has been obtained. When the BTC is equal to $c_{i}$, the dynamic training data set can be expressed as

(9) $$D^{\left(c_{i}\right)}=\left\{\left(n_{1},u_{1}^{\left(c_{i}\right)}\right),\left(n_{2},u_{2}^{\left(c_{i}\right)}\right),\left(n_{3},u_{3}^{\left(c_{i}\right)}\right),\cdots \left(n_{N},u_{N}^{\left(c_{i}\right)}\right)\right\},$$

where i=1,2,3,⋯k, and then the regression strategy is

(10) $$\hat{x}=\arg\;\min_{x}f\left(x\right)\equiv \arg\;\min_{x}\sum_{i=1}^{N}{\left[u_{i}^{\left(c_{i}\right)}-v\left(n_{i},x\right)\right]}^{2}.$$

The vector $x={\left[u_{s},u_{r},\tau \right]}^{T}$ contains all the characteristic parameters. Here, the LM algorithm [35] can be used to solve the extreme value of Equation (10).

### 2.3. Dynamic Training Data Set Obtained by Experiments

**Figure 3:**
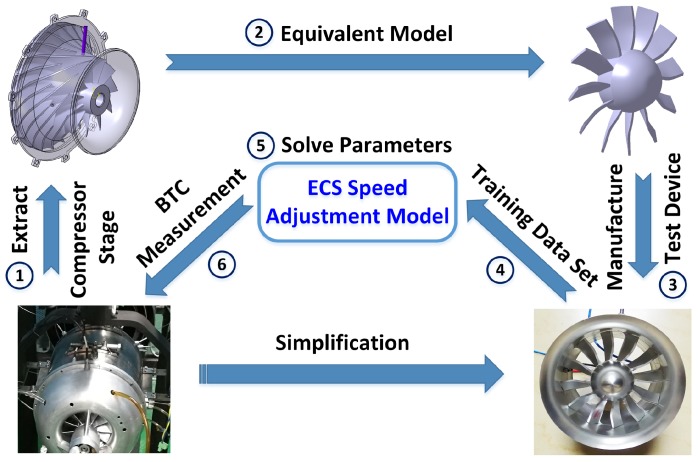
Scheme to obtain the dynamic training data set for training the speed adjustment model.

D can be obtained by numerical simulation or experimental data. However, in practice, it is difficult to accurately simulate the sensor signals due to some uncertainty factors, such as the sensor coil winding and its package manufacturing process, the machining accuracy of the tip surface and the circuit design. Therefore, this paper adopts the experimental method (Figure 3, Step 1 to 6) to obtain the dynamic training data set D under different BTC conditions.

ECS is applied to measure the inlet section of centrifugal compressor in a turbojet engine. Many factors affect the rotor system vibration in normal operation of turbojet engine, and the BTC of centrifugal compressor inlet section cannot be controlled as required. In order to obtain accurate and comprehensive dynamic training data set D during the engine test, the compressor needs to be separated from the engine and test separately. Meanwhile, in order to facilitate driving, the centrifugal compressor is made into an equivalent model (Figure 3, which keeps the blade shape, material and surface treatment consistent with the original compressor, ensuring that the SPA of ECS under the same BTC is the same as the original engine environment). The equivalent device includes the permanent magnet brushless motor, the outer cover, ECS, and the equivalent model of compressor. The finite element model of the rotor system [36] based on the Timoshenko beam element is used to analyze the rotor system [37,38,39]; the results show that the first critical speed of the rotor system is 200,000 r/min, which far exceeds the subsequent tests. The rotor has been well balanced, the maximum radial vibration during the experiment is 0.3 g (g = 9.8 m/s^2^), so its lateral vibration can be ignored.

**Figure 4:**
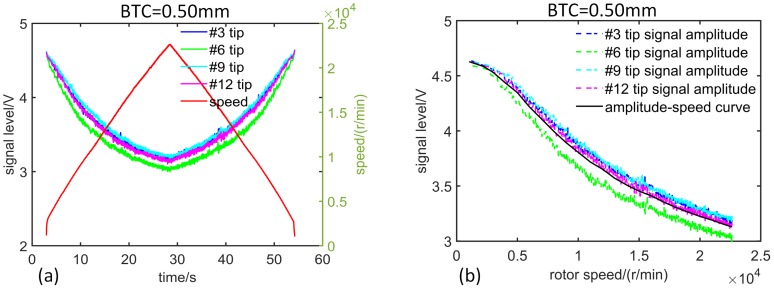
Test results of speed adjustment: (**a**) SPA versus speed in acceleration and deceleration; (**b**) SPA versus speed corresponding to various blades.

When the rotor system is static, the clearance between the equivalent model blade tip and the ECS head is adjusted to 0.5 mm (it changes when the turbomachinery rotates at high speed because the blade will be stretched by centrifugal force). The rotor system is slowly accelerated to the specified speed and then similarly slowed down to the initial speed. The output SPA (Figure 4a) of the sensor corresponding to each blade during the two processes is measured. Then, using the same way, when the static BTC is another value, the SPA sequence $u_{i}$ and rotation speed sequence $n_{i}$ of each blade can be obtained, respectively.

There are certain differences in the output SPA sequences of the sensor corresponding to various blades (Figure 4a) due to manufacturing variations. Nevertheless, during the process of rotor acceleration and deceleration, the SPA curves corresponding to various blades are almost the same, indicating that the operation of the acceleration has no influence on signal measurement. The signal amplitude corresponding to each rotation speed can be considered as the measured value at this uniform speed. In addition, the SPA curves corresponding to various blades remain separated and clear, implying that the measurement accuracy is sufficient, and the rotor system has no whirling and is always in the state of synchronous vibration.

**Figure 5:**
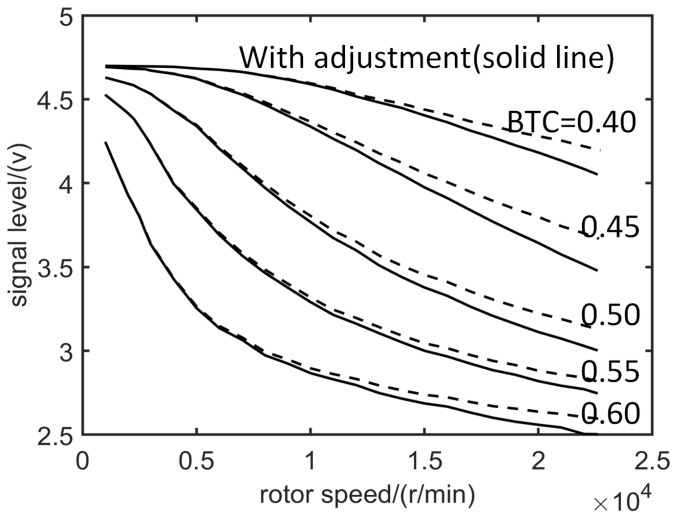
SPA versus speed under various BTCs before (dashed line) and after centrifugal force adjustment (solid line).

The average value of the data which are obtained during the acceleration and deceleration process is calculated to get the sequence of the rotation speed $n_{i}$ and the pulse amplitude output $u_{i}$ of the sensor, corresponding to each blade when the static BTC is 0.5 mm or other value. The output SPA curves of the sensor corresponding to various blades in the time domain are arranged with the rotational speed as the abscissa, so the SPA-speed curves corresponding to various blades are obtained (Figure 4b). The SPA decreases with increasing speed, and the larger the BTC, the faster the amplitude decreases. Taking average of the SPA-speed curves of all blades under each BTC, then the SPA-speed curves (Figure 5, dashed line) under different static BTCs ($c_{i}^{\left(static\right)}$) are obtained.

**Figure 6:**
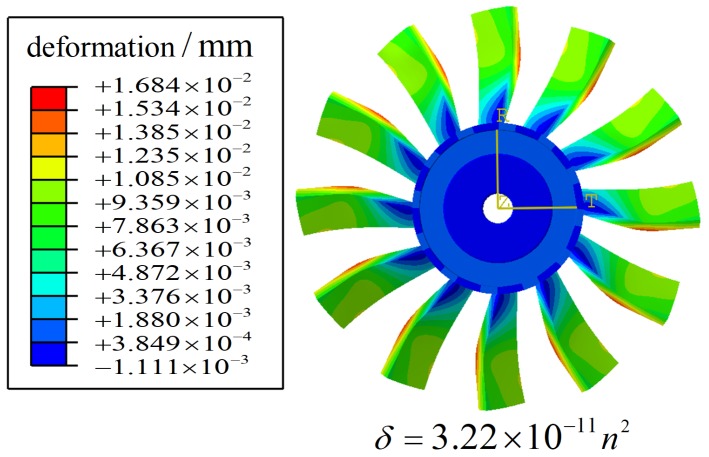
Finite element method (FEM) results of deformation of the equivalent model under centrifugal force.

As mentioned above, the rotating bladed disk will be stretched by centrifugal force. When rotating speed is *n*, the blade tip displacement δ satisfies the relation $\delta \propto n^{2}$[40], let $\delta =k_{\omega }n^{2}$. Use the finite element method (FEM) (Figure 6) to get the result of $k_{\omega }$. The calculation formula of dynamic BTC $c_{i}^{\left(dynamic\right)}$ when the bladed disk rotates is given by

(11) $$c_{i}^{\left(dynamic\right)}=c_{i}^{\left(static\right)}-k_{\omega }n^{2}.$$

The SPA-speed curves of static BTC are corrected with centrifugal force deformation

(12) $$u=f^{int}\left(c^{\left(dynamic\right)},u^{\left(static\right)},c\right),$$

where $f^{int}$ is a spline interpolation [41] function. Then, the final pulse amplitude–speed curves for the different BTCs are obtained (Figure 5, solid line). Thus, the dynamic training data set is obtained by using the method mentioned above.

### 2.4. Calculation of Model Characteristic Parameters

**Figure 7:**
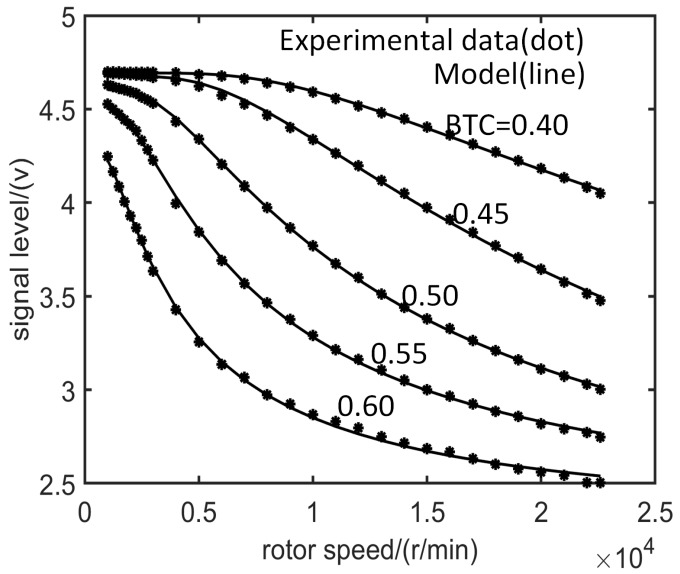
Comparison between experimental data (dot) and theoretical calculation based on SAM (line).

According to the dynamic training data set D obtained by the test, the characteristic parameters of the SAM can be calculated using the LM algorithm. The calculated SAM curves match very well with the test data (Figure 7), and the goodness of fit ($R^{2}$) can reach $R^{2}\geq 0.999$ (Table 1).

**Figure 8:**
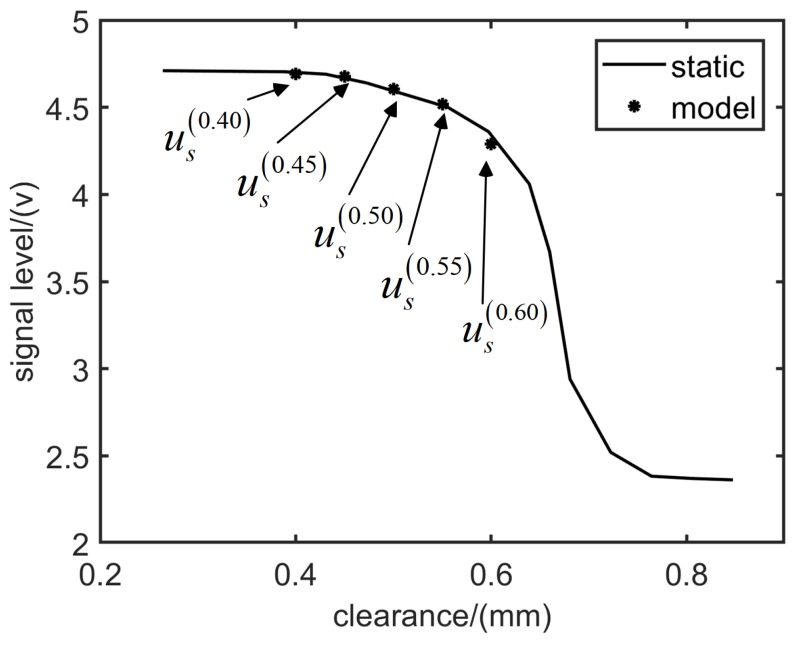
Comparison between engine static test data and $u_{s}$ obtained from SAM.

The characteristic parameter $u_{s}$ in the SAM indicates the SPA value as $t_{m}$ approaches ∞. It also matches quite well (Figure 8) with the sensor characteristic curve measured when the engine rotor is static ( it means that $t_{m}$ is large enough ).

The characteristic parameters τ means response time of electromagnetic interaction between ECS and tip. The response frequency of ECS is related to BTC. As shown in Table 1, τ increases rapidly with increasing BTC, partial derivative of τ with respect to BTC (∂τ/∂c) increases with increasing BTC. When BTC changes from 0.4 mm to 0.6 mm, the response time becomes nine times. When the BTC is equal to 0.5 mm, the speed varies from 0 to 20,000 r/min, and the SPA is reduced to 67%.

The measurement accuracy of ECS is related to both BTC and speed range (Figure 7). When the speed is less than 5000 r/min, partial derivative of SPA with respect to BTC (∂u/∂c) increases with increasing BTC (Table 1), it means that SPA reflects more sensitively to BTC change with increasing BTC. When the speed is higher than 15,000 r/min, the signal amplitude reflects less sensitively to BTC change with increasing BTC. These are significant references to designing appropriate sensors according to the measured object.

## 3. BTC Measurement and Geometric Evaluation Method

In this section, firstly, a method to construct a set of the SPA-speed curves based on the SAM is introduced. Then, based on spline interpolation, we calculate BTC according to the experimental data. Next, the geometric constraint equation of the BTC distribution corresponding to all blades when the rotor deviates from the vibration equilibrium position is established. Finally, a method based on the equation to solve the shaft position vector and evaluate the accuracy of the measured BTC data is presented.

### 3.1. BTC Measurement Method

**Figure 9:**
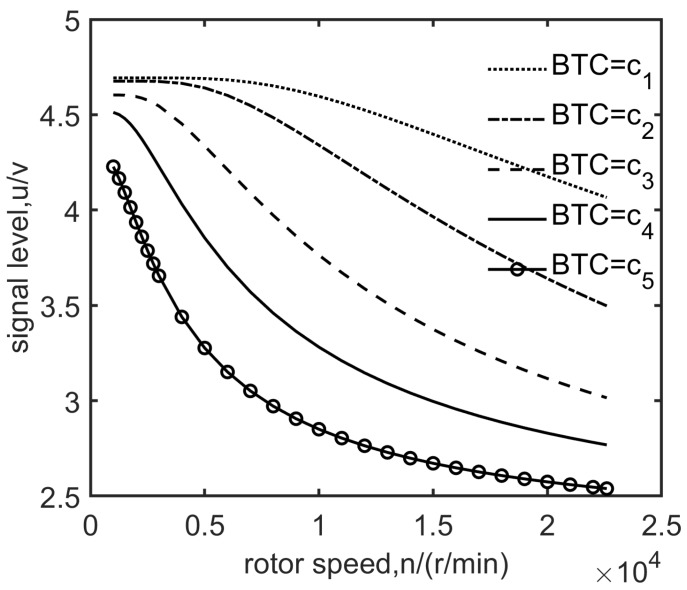
SPA versus speed under different BTCs.

As mentioned in Section 2, when the BTC is a certain value $c_{i}$, the dynamic training data set D can be obtained by experiments; then, the value of $x={\left[u_{s},u_{r},\tau \right]}^{T}$ of the characteristic parameter in u=v(n,x) can be calculated by nonlinear regression. Given a monotonically increasing sequence of BTC as $c=\left\{c_{1},c_{2},c_{3},\ldots ,c_{k}\right\}$, we can get a set of SPA-speed curves (Figure 9).

(13) $$G:u=\left[v\left(n,x^{\left(c_{1}\right)}\right),v\left(n,x^{\left(c_{2}\right)}\right),\ldots ,v\left(n,x^{\left(c_{k}\right)}\right)\right].$$

Assuming that a signal amplitude of a blade in this experiment is $u_{test}$, the measured speed of rotor is $n_{test}$. According to the definition of G, when the rotational speed is $n_{test}$, the signal amplitude sequence $u\left(n_{test}\right)$ corresponding to all the BTC values $c=\left\{c_{1},c_{2},c_{3},\ldots ,c_{k}\right\}$ can be obtained,

(14) $$u\left(n_{test}\right)=\left[v\left(\left(n_{test}\right),x^{\left(c_{1}\right)}\right),v\left(\left(n_{test}\right),x^{\left(c_{2}\right)}\right),\ldots ,v\left(\left(n_{test}\right),x^{\left(c_{k}\right)}\right)\right].$$

Then, the BTC value $c_{x}$ corresponding to the SPA $u_{test}$ satisfies

(15) $$\frac{1}{u_{test}}\left|f^{int}\left(c,u\left(n_{test}\right),c_{x}\right)-u_{test}\right|<\varepsilon .$$

ε is a given relative precision ($10^{-6}$). Then, the corresponding BTC value $c_{x}$ of the blade can be solved.

### 3.2. Geometric Evaluation Method

**Figure 10:**
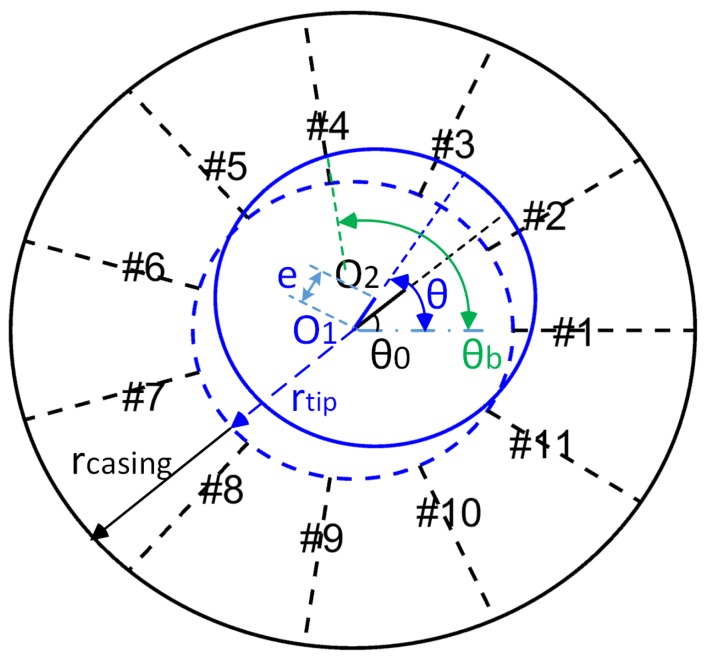
Geometric distribution of BTC.

When the rotor system is running at high speed, the turbomachinery vibrates laterally due to mass imbalance or other non-axisymmetric forces. With the establishment of the polar coordinate system attached to a rotor shaft, the rotor equilibrium position $O_{1}$ is taken as the origin; the first blade is taken as 0-pole angular position, and the phase of the mass imbalance force is denoted as $\theta_{0}$ (Figure 10). Assuming that the rotor has no lateral vibration, the trajectory of the blade tips is a circle, with $O_{1}$ as the origin and $r_{tip}$ as the radius. The inner wall of the casing is a circle, with radius of $r_{casing}$, so $r_{casing}$-$r_{tip}$ is BTC. In general, the BTC is approximately equal to one percent of $r_{tip}$. When the rotor exhibits lateral vibration, the phase of the vibration is $\theta_{0}$, and the amplitude is *e*. At this time, the position of the rotating shaft is $O_{2}$. Thus, the amount of BTC reduction on the *i*-th blade position can be expressed as

(16) $$\Delta d_{i}^{\left(geo\right)}=e\cos\left(\frac{2\pi i}{n_{b}}-\frac{2\pi }{n_{b}}-\theta \right).$$

Mark the BTC measurement value corresponding to the i-th blade as $c_{i}^{\left(m\right)}$, and the reduction of the BTC corresponding to the *i*-th blade due to the rotor lateral vibration calculated from the experimental data can be expressed as:

(17) $$\Delta d_{i}^{\left(m\right)}=\frac{1}{n_{b}}\sum_{i=1}^{n_{b}}c_{i}^{\left(m\right)}-c_{i}^{\left(m\right)}.$$

The BTC reduction of the i-th blade $\Delta d_{i}^{\left(geo\right)}$ calculated from the geometric constraint equation should be equal to $\Delta d_{i}^{\left(m\right)}$ obtained from the experimental data. Therefore, the measured data require satisfying the geometric constraint equation

(18) $$\frac{1}{n_{b}}\sum_{i=1}^{n_{b}}c_{i}^{\left(m\right)}-c_{i}^{\left(m\right)}-e\cos\left(\frac{2\pi i}{n_{b}}-\frac{2\pi }{n_{b}}-\theta \right)=0.$$

Let the rotor axis position vector be p=(e,θ). Then, it is estimated by

(19) $$\hat{p}=\arg\;\min_{p}f\left(p\right)\equiv \arg\;\min_{p}\sum_{i=1}^{n_{b}}{\left[\frac{1}{n_{b}}\sum_{i=1}^{n_{b}}c_{i}^{\left(m\right)}-c_{i}^{\left(m\right)}-e\cos\left(\frac{2\pi i}{n_{b}}-\frac{2\pi }{n_{b}}-\theta \right)\right]}^{2},$$

in order to solve the SAM characteristic parameters, and the LM method can also be used to solve $\hat{p}$. In addition, $R_{p}^{2}$ (Goodness of Fit) is used to evaluate the measurement accuracy of BTC:

(20) $$R_{p}^{2}=1-\frac{\sum_{i=1}^{n_{b}}{\left[\frac{1}{n_{b}}\sum_{i=1}^{n_{b}}c_{i}^{\left(m\right)}-c_{i}^{\left(m\right)}-\hat{e}\cos\left(\frac{2\pi i}{n_{b}}-\frac{2\pi }{n_{b}}-\hat{\theta }\right)\right]}^{2}}{\sum_{i=1}^{n_{b}}{\left[c_{i}^{\left(m\right)}-\frac{1}{n_{b}}\sum_{i=1}^{n_{b}}c_{i}^{\left(m\right)}\right]}^{2}}.$$

## 4. Engine Test

**Figure 11:**
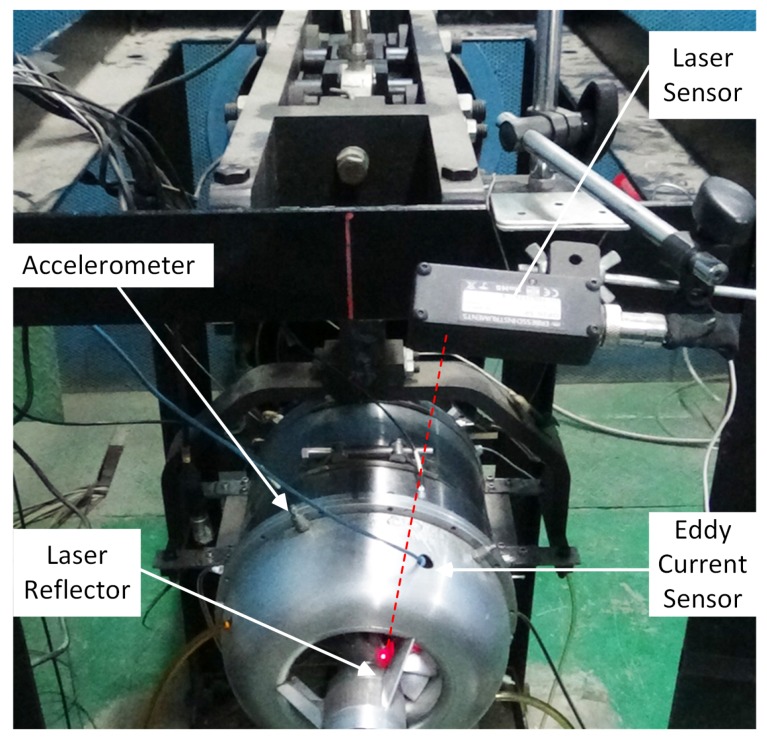
Turbojet engine test rig.

**Figure 12:**
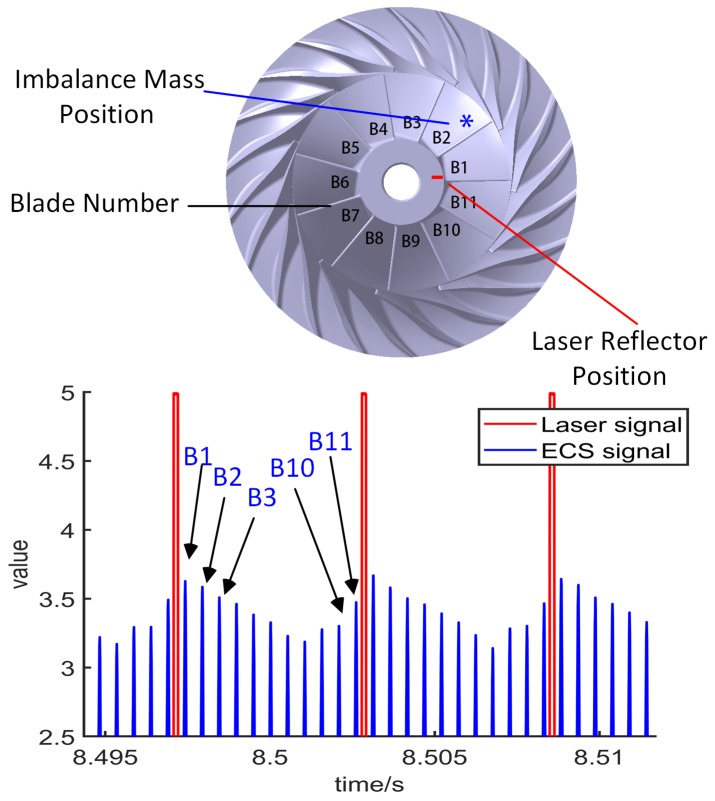
Compressor phase configuration.

The aforementioned method of measuring BTC using ECS is applied to measure the BTC of the inlet section of the centrifugal compressor (Table 2), and the geometric constraint equation is used to evaluate the measured data during the turbojet engine running process. The test of measuring BTC using ECS is performed on the turbojet engine test rig (Figure 11). The point to be measured locates at the inlet section of the centrifugal compressor, with a radius of about 60 mm. In order to make the measurement results more obvious, without dynamic-balance adjustment for the compressor side, and the mass imbalance is set to be five times the allowable value specified in the standards, whose phase is 40° (Figure 12, make the No. 1 blade position be 0° phase). The laser-reflecting patch is mounted on the 0° phase. The laser sensor sends a pulse signal every one revolution of the engine to establish a one-to-one correspondence between the ECS output pulse and the compressor blade.

**Figure 13:**
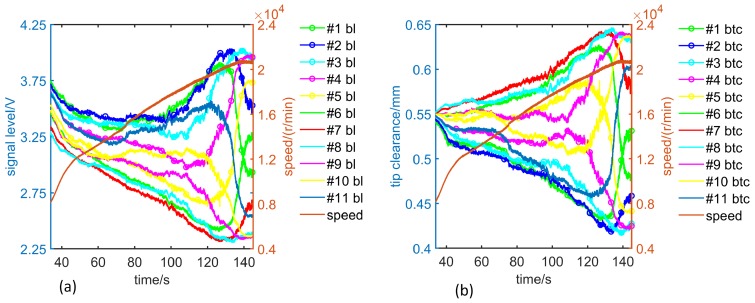
ECS signal and BTC: (**a**) SPA data corresponding to various blades collected during the engine test; (**b**) BTC of various blades during the engine test.

A segment of the sensor output signal data is analyzed here. The engine running speed ranges from 8000 r/min to 21,000 r/min in this section. The SPAs of ECS signal corresponding to various blades are extracted (Figure 13a), and the BTC values of various blades are solved using the SAM (Figure 13b). When the engine runs between 8000 r/min and 16,000 r/min, the relative position of BTC curves corresponding to various blades are almost unchanged. No. 1, No. 2 and No. 3 blades correspond to smaller BTCs whose values decrease with increasing speed, while No. 7 and No. 8 blades correspond to larger BTCs whose values increase with increasing speed. The deflection direction of the rotor points to No. 2 blade, which is consistent with the phase of the mass imbalance force (phase 40°, Figure 12). Because the aerodynamic force is small at low speed, the lateral vibration of the rotor system is synchronized with the rotor mass imbalance force. When the engine speed is above 20,000 r/min, the BTC curves corresponding to various blades are obviously staggered, and the lateral vibration of the rotor system is no longer synchronized with the rotor mass imbalance force, which leads to low frequency whirl.

**Figure 14:**
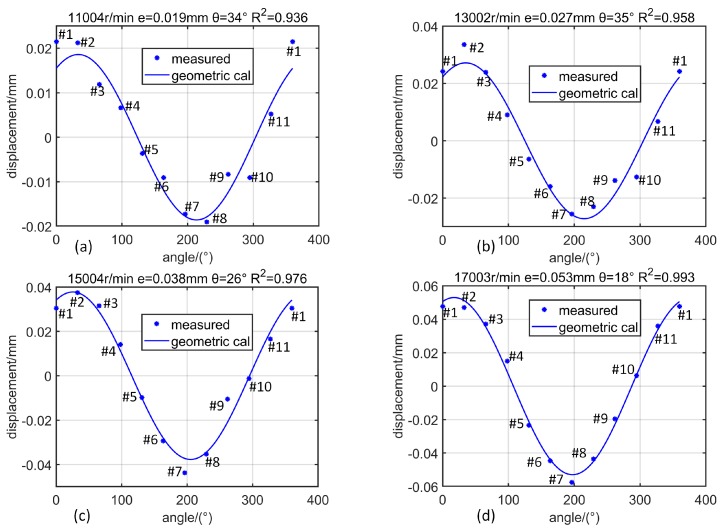
Distribution of BTC obtained from measured data and geometric equation calculation corresponding to different rotational speeds: (**a**) 11,000 r/min; (**b**) 13,000 r/min; (**c**) 15,000 r/min; (**b**) 17,000 r/min.

**Figure 15:**
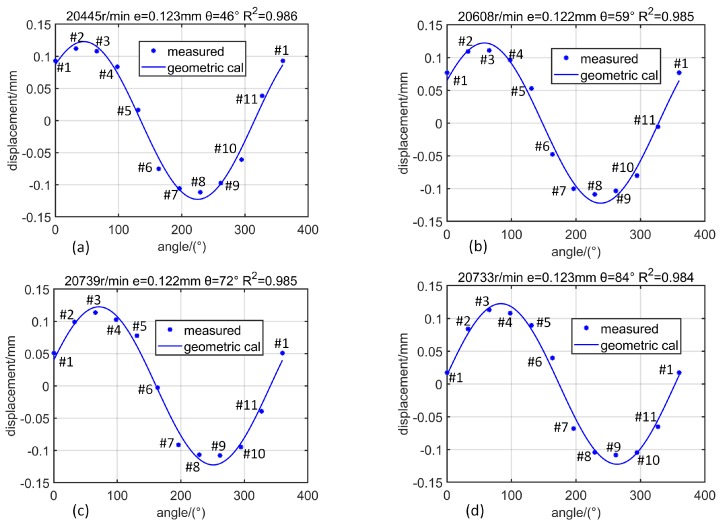
Distribution of BTC obtained from measured data and geometric equation calculation corresponding to different rotor vibration phases when the rotational speed remains about 20,500 r/min: (**a**) θ=46°; (**b**) θ=59°; (**c**) θ=72°; (**d**) θ=84°.

After having solved the BTCs corresponding to various blades of the compressor, the shaft position vector p=(e,θ) can be parsed according to the geometric constraint equation. Firstly, as the rotational speed increases (Figure 14, from 11,000 r/min to 17,000 r/min), the shaft lateral vibration amplitude *e* gradually increases, and $R_{p}^{2}$ gradually approaches 1. At a lower speed of 11,000 r/min (Figure 14a), the shaft lateral vibration amplitude is 0.019 mm. At this time, there is a significant error ($R_{p}^{2}=0.936$) between the measured data and the geometric constraint equation. At a higher speed of 17,000 r/min (Figure 14d), the shaft lateral vibration amplitude is 0.053 mm, and the measured data matches very well with the geometric constraint equation ($R_{p}^{2}=0.993$). Then, when the rotor reflects a slight whirl and the rotational speed is almost unchanged (Figure 15, 20,500 r/min), the rotor vibration amplitude varies between 0.122 and 0.123 mm while the phase changes from 46° to 84° (46°, 59°, 72°, 84°), the measured data and the geometric constraint equation matches very well ($R_{p}^{2}>0.98$). Therefore, it can be considered that the accuracy of BTC measurement is 0.01 mm.

## 5. Conclusions

BTC measurement technology is one of the key technologies in engine health management and BTC active control. This article focuses on BTC measurement using ECS; on the basis of elaborating the principle of measuring BTC using ECS, a speed adjustment model (SAM) is proposed, and the measurement method of BTC is developed based on this model. Combined with the structural characteristics of turbomachinery, a method for evaluating measurement results by using a geometric constraint equation is proposed. The key results are summarized as follows:

- The relationship between response time of the ECS measurement loop and BTC is quantitatively studied by an experimental method, and response time increases rapidly with increasing BTC. When BTC changes from 0.4 mm to 0.6 mm, the response time becomes nine times. The relationship between the output signal and the rotational speed is also quantitatively studied. As the speed increases, the signal amplitude decreases. When the BTC is equal to 0.5 mm, the speed varies from 0 to 20,000 r/min, the SPA is reduced to 67%.
- A high-precision SAM for measuring BTC using ECS is proposed. The characteristic parameters of the model are solved by the experimental method. The goodness of fit between the SAM and the dynamic training data set is greater than 0.999. The explicit physical meaning of the model characteristic parameters is given, which is of great significance for the rational design and accurate usage of ECS.
- A BTCMM based on the SAM is proposed and applied to the BTC measurement of the compressor inlet during the engine test. The experimental results show that this method has the repeatability precision of 0.01 mm in the actual test.

## Figures and Tables

**Table 1:** Characteristic parameters obtained from the dynamic training data set.

Blade Tip Clearance (BTC)/mm	$u_{s}$/*V*	τ/($10^{-4}$ s)	$R^{2}$	∂τ/∂c	∂u/∂c (n = 5000)	∂u/∂c (n = 15,000)
0.40	4.693	0.1422	0.9990	1.7319	–1.5112	–7.9378
0.45	4.676	0.2119	0.9991	2.8799	–3.6381	–11.5880
0.50	4.604	0.4177	0.9997	5.5075	–8.4215	–9.8306
0.55	4.517	0.7667	0.9997	9.2653	–10.9046	–7.5369
0.60	4.289	1.3590	0.9990	15.573	–12.1230	–7.3601

**Table 2:** Engine test conditions.

Item	Specification
Turbojet engine	Compressor inlet-11 full blades, static BTC = 0.55 mm
Eddy current sensor	Response frequency: 25 kHz
Laser sensor	Response frequency: 5 kHz
Accelerometer	50 mV/g
Data acquisition device	2.5 MS/s

## Abbreviations

BTC	blade tip clearance
ACC	active clearance control
ECS	eddy current sensor
SPA	signal pulse amplitude
SAM	speed adjustment model
BTCMM	BTC measurement method
